# Primary pleuropulmonary and mediastinal synovial sarcoma: a clinicopathologic and molecular study of 26 genetically confirmed cases in the largest institution of southwest China

**DOI:** 10.1186/s13000-016-0513-3

**Published:** 2016-07-11

**Authors:** Ting Lan, Huijiao Chen, Bo Xiong, Tingqing Zhou, Ran Peng, Min Chen, Feng Ye, Jin Yao, Xin He, Yaqin Wang, Hongying Zhang

**Affiliations:** Department of Pathology, West China Hospital, Sichuan University, Guoxuexiang 37, 610041 Chengdu, Sichuan China; Department of Pathology, Mianyang People’s Hospital, Mianyang, Sichuan China; Laboratory of Pathology, West China Hospital, Sichuan University, Chengdu, Sichuan China; Department of Radiology, West China Hospital, Sichuan University, Chengdu, Sichuan China; Department of Pathology, Affiliated Hospital of North Sichuan Medical College, Nanchong, Sichuan China

**Keywords:** Synovial sarcoma, Pleuropulmonary, Mediastinum, *SS18-SSX* fusion transcript, Prognosis

## Abstract

**Background:**

Primary pleuropulmonary and mediastinal synovial sarcomas (PPMSSs) are extremely rare. The authors present the largest series in an Asian population.

**Methods:**

Between 2000 and 2015, 26 genetically confirmed PPMSSs were included. The clinicopathologic features of all of the cases were reviewed. Immunohistochemical staining was carried out using the following antibodies: TLE1, cytokeratin (AE1/AE3), EMA, CD99, Bcl-2, CK7, CD34, S-100 protein, and Ki-67. The chromosomal translocation t(X;18)(p11.2;q11.2) was detected by fluorescence in situ hybridization (FISH) and reverse transcription polymerase chain reaction (RT-PCR). We compared the clinical, pathologic, immunohistochemical, and molecular features of this series with that of the previous series and soft tissue synovial sarcomas.

**Results:**

This series included 17 males and nine females. The median age was 36.5 years (range, 16–72 years). The tumors involved the lung (76.9 %), pleura (15.4 %), and mediastinum (7.7 %). The median tumor size was 6 cm (range 2.3 ~ 24 cm). The majority of the tumors were well-circumscribed. The tumors were classified as monophasic (84.6 %), biphasic (3.8 %), and poorly differentiated (11.5 %) types. The tumors were graded as French Federation of Cancer Centers (FNCLCC) grade 2 (62.5 %) and FNCLCC 3 (37.5 %). Diffuse immunostaining for TLE1, BCL-2, and CD99 was identified in 91.7, 95.7, and 56.0 % of the tumors, respectively. Focal positivity was seen with EMA (84.6 %), CK7 (55.6 %), cytokeratin (AE1/AE3) (68.0 %), CD34 (5.0 %), and S-100 protein (21.7 %). A high Ki-67 index (≥10 %) was observed in 91.3 % of the tumors. The fusion transcripts included *SS18-SSX1* (15/22, 68.2 %), *SS18-SSX2* including variants (6/22, 27.3 %)*,* and *SS18-SSX4* (1/22, 4.5 %) fusions. The remaining four cases showed positivity for *SS18* rearrangement by FISH. Surgical excision of tumors or lobectomy were performed in 20 patients, and seven of the patients underwent adjuvant therapy. Clinical follow-up was available in 73.1 % cases, with a median follow-up of 12.0 months. The median survival time was 14.5 months. Tumor resection (*p* = 0.024) and no residual tumor (*p* = 0.004) were associated with an improved overall survival time.

**Conclusions:**

PPMSS is a highly aggressive neoplasm. Extensive surgical resection of the tumor and more effective adjuvant therapy should be advocated. PPMSS must be differentiated from similar diseases.

## Background

Synovial sarcoma (SS) is a morphologically, clinically, and genetically well-defined soft-tissue neoplasm. SSs are characterized by the t(X;18)(p11.2;q11.2) translocation, which leads to *SS18-SSX* gene fusion [1, 2], and extremely rare neoplasms harbor *SS18L1-SSX1*, resulting from t(X;20) [3, 4]. Most tumors occur in the extremities near the joints, followed by the trunk and head and neck regions [1, 2]. Primary SSs have also been reported to arise from a variety of unusual locations, such as the mediastinum, retroperitoneum, and various viscera [1, 2].

Primary pleuropulmonary and mediastinal SSs (PPMSSs) are extremely unusual and have gradually been recognized as a clinicopathological entity [4, 5] since this type of peculiar tumor has been described in the literature over the past 26 years [6]. A diagnosis of PPMSS is difficult for clinicians and pathologists because of the rare nature of the tumor.

To the best of our knowledge, there have been 13 series consisting of five or more cases of PPMSSs in the English literature [7–19], and only seven of these studies were genetically confirmed. Among these t(X;18)(p11.2;q11.2)-positive series, five of them were confirmed by reverse transcription polymerase chain reaction (RT-PCR) [8, 12–14, 16], and the remaining two series were confirmed by fluorescence in situ hybridization (FISH) [15] and classical cytogenetics [11], respectively.

To better understand the clinicopathologic spectrum of these rare lesions, the authors describe a series of 26 genetically confirmed PPMSSs at one of the largest medical centers in China. This series is the third largest in the English literature and is the largest population-based analysis in an Asian population.

## Methods

### Patients

This study was approved by the West China Hospital Institutional Review Board. A SNOMED search of the hospital surgical pathology files from January 2000 to May 2015 identified 489 spindle cell tumors located in the pleuropulmonary and mediastinal area, whereas 24265 lung carcinomas were detected during this period. The tumors were reviewed by two pathologists with soft tissue tumor pathology expertise (H.Z. and H.C) and three general surgical pathologists (T.L., B.X., and T.Z). Two hundred and seventy-seven of the spindle cell tumors were sarcomas. Eighty-seven metastatic sarcomas (including 28 SSs) were excluded, and 190 of 277 spindle cells sarcomas were primary tumors. SS was the most common primary sarcoma (28/190, 14.7 %) in this location, followed by liposarcoma (14/190, 7.4 %), Ewing sarcoma (13/190, 6.8 %), leiomyosarcoma (13/190, 6.8 %), and chondrosarcoma (11/190, 5.8 %). Finally, 26 t(X;18)(p11.2;q11.2) -positive PPMSSs were included in this study.

### Radiology methods

All the available images were reviewed by a radiologist with thoracic and oncologic imaging expertise (J.Y.). Several parameters were evaluated, including the margin, pleural effusion, mediastinal shift, tumor heterogeneity, enhancement, presence of calcification, necrosis/cystic change and lymphadenopathy.

### Histologic evaluation

The tumors were classified into monophasic, biphasic, and poorly differentiated types according to the criteria proposed by the World Health Organization [1] and Enzinger and Weiss's Soft Tissue Tumors [2]. Grading was performed following the French Federation of Cancer Centers (FNCLCC) grading system [20].

### Immunohistochemistry

Immunohistochemical staining was carried out on formalin-fixed paraffin-embedded (FFPE) tissue, using the EnVision Plus detection system (DAKO, Carpinteria, CA) with controls. Standard immunohistochemical studies were performed using the following antibodies: TLE1, cytokeratin (AE1/AE3), EMA, CD99, Bcl-2, CK7, CD34, S-100 protein, and Ki-67 (Table 1). The staining intensity of TLE1 was graded as “3+”, “2+”, “1+” or “-” (negative) according to Terry et al. [21]. The index of Ki-67 ≥ 10 % was considered high [22].

**Table 1:** Antibodies performed and subcellular distribution

Antibody	Laboratory	Clone	Dilution	Subcellular distribution
TLE1	Santa Cruz Biotechnology, USA	polyclonal M-101	1:100	Nucleus
cytokeratin	Dako, Carpintera CA	monoclonal AE1/AE3	1:200	Cytoplasm/Membrane
EMA	Dako, Carpintera CA	monoclonal E29	1:100	Cytoplasm/Membrane
CD99	Dako, Carpintera CA	monoclonal 12E7	1:100	Cytoplasm/Membrane
Bcl-2	Dako, Carpintera CA	monoclonal 124	1:200	Membrane
CK7	Dako, Carpintera CA	monoclonal OV-TL 12/30	1:100	Cytoplasm
CD34	Buffalo Grove, IL	monoclonal QBEnd/10	1:50	Cytoplasm
S-100 protein	Dako, Carpintera CA	polyclonal anti-S-100	1:400	Cytoplasm
Ki-67	Dako, Carpintera CA	monoclonal MIB-1	1:100	Nucleus

When staining for TLE1, 39 nonsynovial sarcomas were selected as the control samples, including ten solitary fibrous tumors (SFTs), six spindle cell carcinomas, ten type A thymomas, ten malignant mesotheliomas, and three type I pleuropulmonary blastomas.

### Fluorescence in situ hybridization (FISH)

All the 26 tumors had material available for FISH studies. We used a commercially available Vysis LSI SS18 Dual Color Break Apart Probe (Abbott Molecular, Des Plaines, IL, USA) for *SS18* on chromosome 18q11.2. The first probe was labeled in spectrum orange (telomeric, 5’ to *SS18*, 650 Kb), whereas the second probe was labeled in spectrum green (centromeric, 3’ to *SS18*, 1040 Kb). The FISH analyses were performed according to the manufacturer-provided protocol. FISH assays were carried out on 4-μm-thick FFPE tissue sections. The sections were deparaffinized in xylene twice for 30 min, and dehydrated in 100 % ethanol twice for 5 min. The sections were pretreated using the Paraffin Pretreatment Kit (Vysis). Tissue sections were then digested with Digest All-3 (Zymed, San Francisco, CA, USA) twice for 5 min. Tissue sections were denatured at 83 °C for 5 min and hybridized overnight at 42 °C in a humidified chamber. The slides were then washed with 0.3 % NP40/2 × SSC at 73 °C for 2 min, and again with 0.1 % NP40/2 × SSC at room temperature for 2 min. Slides were counterstained with 2.0 μg/ml of 4',6-diamidino-2-phenylindole (Abbott Molecular, Des Plaines, IL, USA). Tumor samples were scored by two investigators in 100 cells in a blind fashion in each case. A split signal pattern was considered positive for the gene rearrangement if the distance between the green and the red signals were greater than the diameter of any two signal. A case was considered positive for rearrangement when 10 % or more of the cells showed split apart signals.

### Reverse transcription polymerase chain reaction (RT-PCR)

Using reverse transcriptase polymerase chain reaction (RT-PCR) analysis, 23 cases with enough material were analyzed for the presence of the fusion genes *SS18-SSX1*, SS18*-SSX2*, and SS18*-SSX4*. Total RNA was isolated from 10-μm sections of FFPE tissue material using the High Pure FFPE RNA Micro Kit (Qiagen, Valencia, CA), according to the manufacturer’s instructions. To eliminate the contamination of genomic DNA, RNA samples were treated with DNase I. RNA reverse-transcription into cDNA was performed using the Transcriptor First Strand cDNA Synthesis kit (Roche Applied Science, Indianapolis, IN) for 1 h at 42 °C and 5 min at 85 °C using SSX-1/2-b reverse primer: 5’CATTTTGTGGGCCAGATGC3’. All polymerase chain reactions (PCRs) were performed for 30 cycles using the GoTaq DNA polymerase with GoTaq green buffer (Promega, Madison, WI) with the following cycle conditions: denaturation at 94 °C for 7 minute, annealing at 58 °C for 30 s, and extension at 72 °C for 30 s. The primers were used in the following combinations: *SS18-SSX* consensus forward primer: 5'-agaccaacacagcctggaccac; *SS18-SSX1*–specific reverse primer: 5'-acactcccttcgaatcattttcg; *SS18-SSX2*–specific reverse primer: 5'-gcacttcctccgaatcatttc and *SS18-SSX4*–specific reverse primer: 5'-gcacttccttcaaaccattttct. Normal lung tissue was used as the negative control, and glyceraldehyde-3-phosphated ehydrogenase (GAPDH) as the reference gene. Products of classic fusion gene were 108 bp. The PCR products were gel purified by using the QIAquick Gel Extraction kit (Qiagen, Valencia, CA) and sequenced by using an ABI PRISM 3100 Genetic Analyzer (Applied Biosystems, Foster City, CA).

### Statistical analysis

Summary statistics were obtained using standard methods. Survival analyses were computed by the Kaplan-Meier method. Gender, age at presentation (<37 years old, ≥37 years old), tumor size (<5 cm, ≥5 cm), FNCLCC grade, mitotic rate (<10/10 high power filed, ≥10/10 high power filed), gene fusion type (*SS18-SSX1*, *SS18-SSX2*), tumor resection (yes, no) and residual tumor status (yes, no; assessed microscopically) (residual tumor was defined as treatment without tumor resection or resected tumors with positive margins) were considered to have prognostic value, and the log-rank test was used to compare the two aspects of the different groups. Differences were considered statistically significant when *P* < 0.05. SPSS 22.0 statistical software (IBM Corp, Armonk, NY, USA) was used for all statistical analysis.

## Results

### Clinical findings

The clinical findings are summarized in Table 2. This study included 17 males and nine females (ratios, 1.9:1.0) aged from 16 to 72 years (median, 36.5 years; mean, 37.8 years). The most common symptoms at presentation were cough, chest pain and dyspnea (24/26, 92.3 %), and the remaining two patients were found incidentally. The tumors involved the lung (20/26, 76.9 %), pleura (4/26, 15.4 %), and mediastinum (2/26, 7.7 %). The tumor size ranged from 2.3 to 24 cm (median, 6 cm; mean, 8.5 cm). None of the patients showed any evidence of primary malignancy elsewhere at the time of diagnosis. Smoking information was available for 18 patients, and 38.9 % (7/18) of the patients had a history of smoking.

**Table 2:** Clinical and treatment features of 26 PPMSSs

Case NO.	Age/Gender	Symptoms/preop course at initial Op	Smoking	Size (CM)	Location	Microscopically invasion	Microscopically margin	Lymph node metastasis	Treatment	Progression/interval	Outcome/Follow-up duration
1	M/48 y	Cough, Dyspnea/24 m; Chest pain/1 m	NA	15	RLL	NA	NA	ND	Lobectomy	NA	NA
2	M/52 y	Cough, Expectoration, Blood in phlegm/4 d	NA	3.5	RLL	-	-	ND	Lobectomy	NA	NA
3	F/27 y	Cough, Expectoration/1 m	Never	5	RLL	pleura	-	-	Lobectomy, Lymph node dissection, Chemotherapy (EADM + IFO + DTIC)	NA	NA
4	F/42 y	Chest pain, Hemoptysis/6 m	Never	13	RMLL	pleura	-	-	Lobectomy, Lymph node dissection	NA	NA
5	M/30 y	Cough, Chest pain/15 d	10 y	24	LP	ND	ND	ND	Chemotherapy (IFO + EADM + DDP)	NA	NA
6	M/27 y	Cough, Chest pain, Dyspnea/6 m; Blood in phlegm/1 w	Never	15	LLUL	ND	ND	ND	Biopsy only	Local lymph node metastasis/at presentation	DOD/11 m
7	F/53 y	Cough, Hemoptysis/6 m	Never	6	LUL	-	-	-	Lobectomy, Lymph node dissection, Chemotherapy (CTX + VCR + EADM)	Localized/45 m	DOD/51 m
8	M/20 y	Cough, Chest pain/NA	NA	3	RML	ND	ND	ND	Biopsy only	Intrapulmonary metastasis/at presentation	DOD/8 m
9	F/31 y	Cough, Dyspnea/2 m	NA	15	Anterior mediastinum	NA	NA	ND	Tumorectomy	Localized/6 m	DOD/12 m
10	F/72 y	Cough, Expectoration/3 d	NA	5	RLL	pleura	-	NA	Lobectomy, Lymph node dissection	Intrapulmonary metastasis/29 m	DOD/32 m
11	M/28 y	Cough/24 m; Hemoptysis/2 d	NA	6	LUL	-	-	NA	Lobectomy, Lymph node dissection	NA	NA
12	M/62 y	Cough, Blood in phlegm/3 m	30 y	11.6	LL	ND	ND	ND	Bronchial artery infusion chemotherapy (FudR + DDP + NVB)	Intrapulmonary and thyriod metastasis/at presentation	DOD/15 m
13	F/34 y	Cough, Expectoration, Hemoptysis/1 m	Never	3.5	LLL	-	-	-	Lobectomy, Lymph node dissection, Chemotherapy (IFO + EADM)	Localized/26 m; Left chest wall/36 m;Left rib/39 m; Right lung and spine metastasis/51 m	AWD/52 m
14	M/26 y	Cough, Chest pain, Dyspnea/20 d	7 y	14	LUL	pleura	+	ND	Lobectomy	Localized/8 m	DOD/11 m
15	F/38 y	Cough, Chest pain/NA	NA	8	LUL	NA	NA	ND	Lobectomy	NA	NA
16	F/46 y	Cough, Chest pain/24 m	Never	2.3	RUL	ND	ND	ND	Biopsy only	Brain metastasis/at presentation	DOD/10 m
17	F/39 y	Cough, Chest pain/1 m	Never	10	LUP	Lung; chest wall	-	-	Lobectomy, Lymph node dissection, Chemotherapy (DTIC + EADN)	Adrenal gland metastasis/2 m	DOD/15 m
18	M/46 y	Cough/NA	NA	4.2	LUL	-	-	ND	Lobectomy	Multiple organs metastasis/2 m	DOD/18 m
19	M/20 y	Asymptomatic	Never	3.5	RUP	-	-	ND	Tumorectomy, Chemotherapy (IFO + DDP + EADM), Radiotherapy	Left lung metastasis/24 m	AWD/24 m
20	M/36 y	Asymptomatic	Never	4.5	LLL	-	-	-	Lobectomy, Lymph node dissection	NO	NED/4 m
21	M/16 y	Cough, Hemoptysis/NA	Never	6	RL	pleura	+	ND	Partial tumorectomy	NO	DOD/18 m
22	M/22 y	Chest pain/2 w; Spontaneous hemopneumothorax/1+ w	6 y	5	LP	lung	+	ND	Tumorectomy	NO	DOD/9 m
23	M/47 y	Chest pain, Dyspnea/5 m	30 y	18	RL	ND	ND	ND	Chemotherapy (GT regimen)	NO	AWD/10 m
24	M/60 y	NA	30 y	7	LUL	-	-	-	Lobectomy, Lymph node dissection, Postoperative	Localized/9 m	AWD/9 m
25	M/37 y	Cough, Expectoration,Chest pain, Dyspnea/1 m	10 y	4	LUL	-	-	-	Lobectomy, Lymph node dissection	NO	NED/1 m
26	M/24 y	Dyspnea/3 m;Syncope/2 m	Never	5.3	Valvula bicuspidalis	myocardium	-	ND	Tumorectomy, Chemotherapy	Localized/2 m	DOD/26 m

*Abbreviation*: *NA* not available, *ND* not done, “+” positive, “-” negative. *W* well-defined, *I* ill-defined, *LLL* left lower lobe, *LUL* left uper lobe, *RLL* right lower lobe, *RUL* right uper lobe, *RML* right middle lobe, *RMLL* right middle and lower lobe, *LLUL* left lower and uper lobe, *LP* left pleura, *LUP* left uper pleura, *RUP* right uper pleura, *y* year, *m* month, *w* week, *d* day, *EADM* Epirubicin, *IFO* Ifosfamide, *DTIC* Dacarbazine, *DDP* cis-Dichlorodiamineplatinum, *CTX* cyclophosphamide, *VCR* Vincristine, *NVB* vinorelbine, *FudR* 2'-deoxy-5-fluoro-uridin, *MFSS* monophasic fibrous synovial sarcoma, *BSS* biphasic synovial sarcoma, *PDSS* poorly differentiated synovial sarcoma

Surgical excision of the tumor or a lobectomy were performed in 20 patients (76.9 %). Ten (50 %) of these patients underwent local lymph node dissection, and seven (35 %) received postoperative chemotherapy or radiotherapy. Three of the six inoperable patients were treated with chemotherapy.

### Radiologic findings

Radiology information were available for 23 patients, including ten conventional chest radiographs, 21 computed tomography (CT) images, and two magnetic resonance imaging (MRI) studies.

On the chest radiographs, all the masses were oval or round shaped with sharp or partly defined margins that were homogeneous without calcification or cavities. Ipsilateral pleural effusions were observed in six patients.

**Fig. 1:**
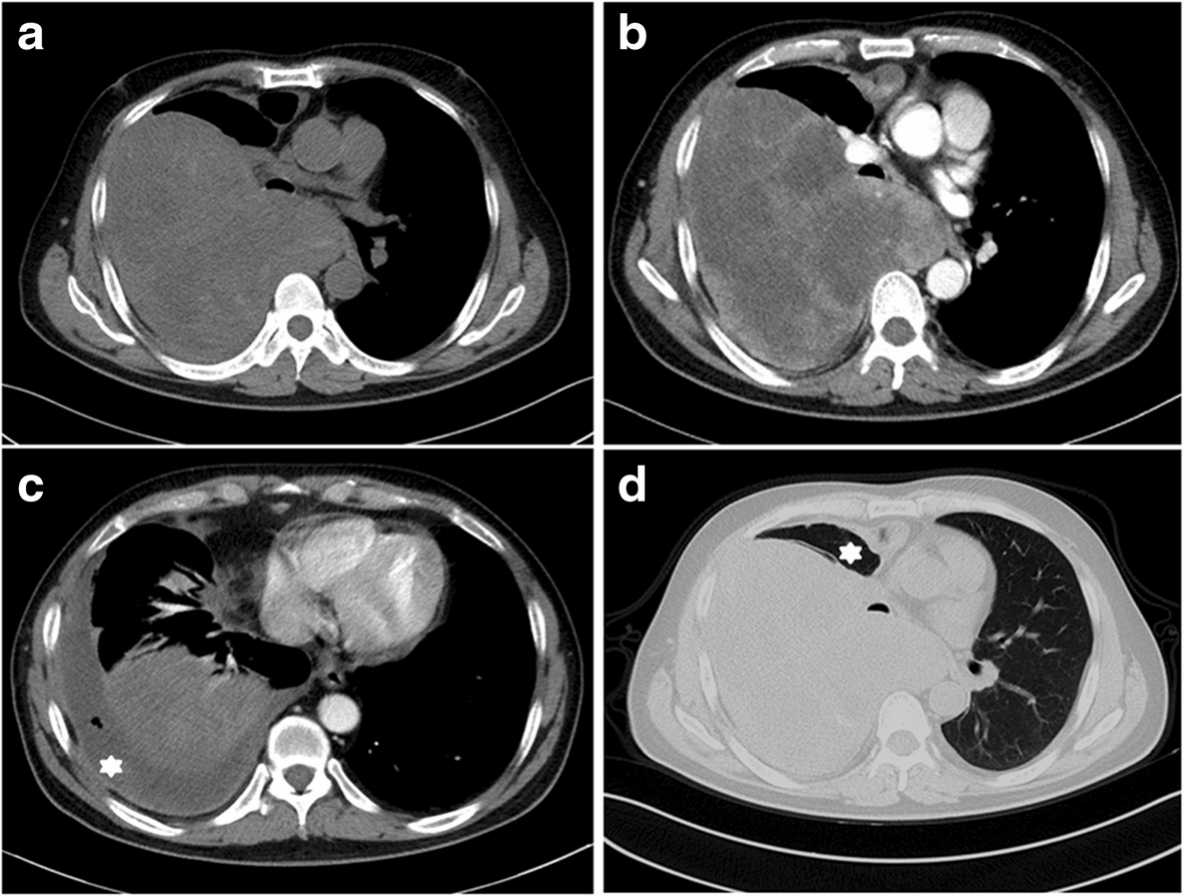
Primary synovial sarcoma of the lung in a 47-year-old male with chest pain and dyspnea. **a** Non-contrast CT (mediastinum window) scan demonstrating a large mass of the right lung, contiguous with the right lateral pleura and the mediastinum, note the mediastinal shift. **b** Contrast-enhanced CT scan (mediastinum window) showing the heterogeneous enhancement mass with predominantly cystic areas and a massive pleural effusion (*white asterisk*, **c**). **d** Contrast-enhanced CT scan (lung window) revealing obvious atelectasis on the right (*white asterisk*)

On CT (Fig. 1), the margins were well-defined in 14/21 (66.7 %) tumors, ill-defined in 1/21 (4.8 %), and infiltrative in 6/21 (28.6 %). Fifteen cases had contrast-enhanced CT data available, and those tumors showed heterogeneous (14/15, 93.3 %) or homogeneous enhancement (1/15, 6.7 %). Necrosis/cystic changes were observed in 11/21 tumors (52.4 %), and calcification was identified in 1/21 (4.8 %). Seven tumors (7/21, 33.3 %) showed lymphadenopathy. Enhancing tumor vessels were identified in two cases. A contralateral mediastinal shift was observed in 7/21 (33.3 %) cases, and ipsilateral pleural effusions were observed in 12/21 (57.1 %) patients.

On MRI, the T1-weighted and T2-weighted images revealed lobulated lesions with sharp margins (2/2). One tumor showed a peripheral rim enhancement after the administration of gadopentetate dimeglumine**.** Cystic areas of hyperintensity suggestive of necrosis, hemorrhage, or myxoid material were observed in one tumor on T2-weighted images.

### Gross and histologic findings

The macroscopic descriptions were available in 18 of the 20 resected specimens. Thirteen (72.2 %) tumors were well-circumscribed and surrounded by thin fibrous pseudocapsules or unencapsulated, and the remaining five (27.8 %) tumors were poorly circumscribed. On cut sections, the tumors were white-gray to tan and soft fleshy to rubbery in texture. Twelve tumors (66.7 %) contained focal necrosis and hemorrhage. Cystic changes were observed in four cases (22.2 %). No grossly visible calcification was observed.

**Fig. 2:**
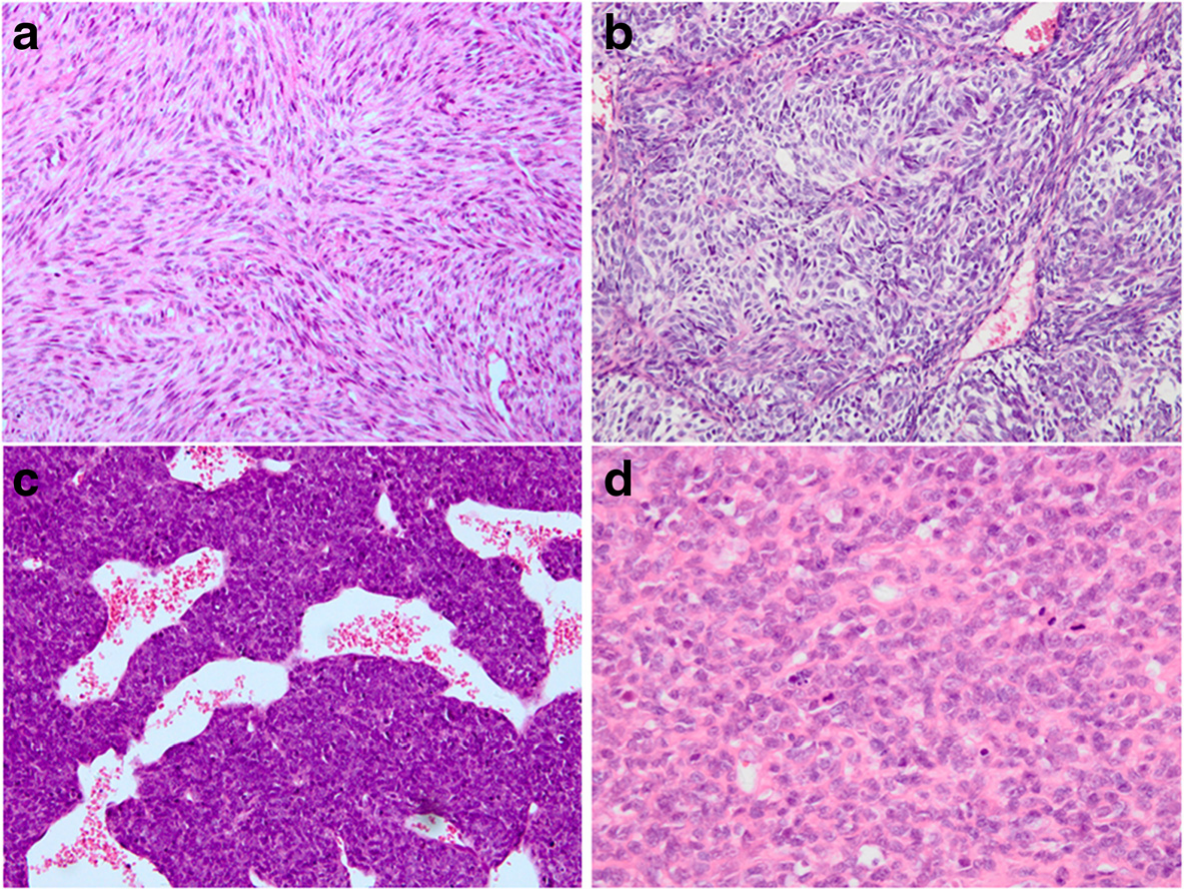
Microscopic features. **a** Monophasic synovial sarcoma, tumor consisting of uniform hyperchromatic spindled cells arranged in a prominent fascicular pattern (H&E, ×200). **b** Biphasic synovial sarcoma, composed of oval epithelial nests and surrounded by spindle cell components (H&E, ×200). **c** Poorly differentiated synovial sarcoma, showing high-grade spindle-shaped cells and a hemangiopericytoma-like vascular pattern, reminiscent of a malignant peripheral nerve sheath tumor (H&E, ×200). **d** Poorly differentiated synovial sarcoma, consisting of large epithelioid cells, indistinguishable from a poorly differentiated carcinoma or mesothelioma (H&E, ×400)

**Fig. 3:**
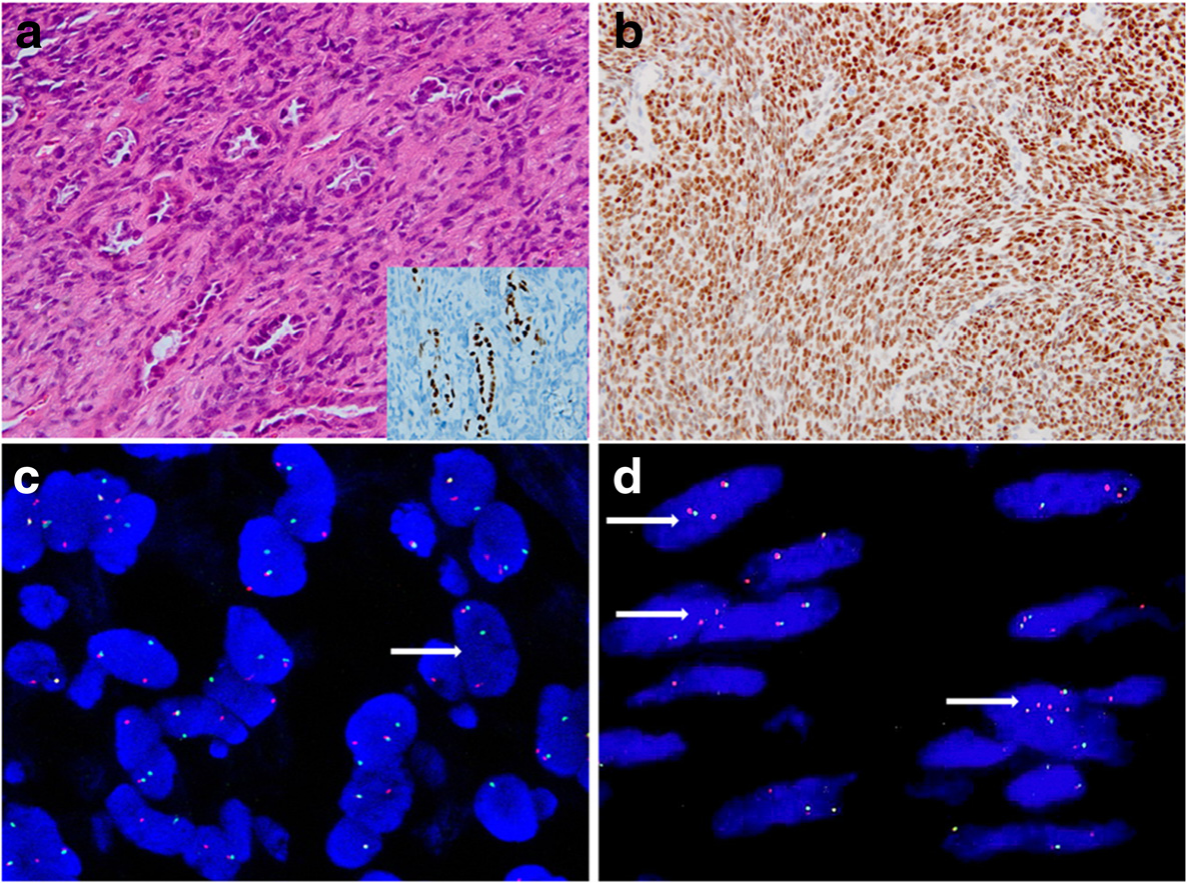
**a** Entrapped pneumocytes. The entrapped lung tissue could be observed in the peripheral areas of the monophasic synovial sarcoma, resembling a biphasic synovial sarcoma. The entrapped pneumocytes are positive for thyroid transcription factor 1 (TTF-1) (a inset, 400×). **b** Tumor cells with “3+” positively nuclear TLE1 expression (×200). **c** FISH demonstrating a balanced rearrangement of the *SS18* locus observed in the majority of the neoplastic cells (×1000). **d** In Case 9, the majority of tumor cells show two paired signals and one or multiple extra red signals, loss of green signal (*arrow*) (×1000)

The histologic findings are summarized in Table 3. The tumors were classified into monophasic SS (MSS) (22/26, 84.6 %), biphasic SS (BSS) (1/26, 3.8 %), and poorly differentiated SS (PDSS) (3/26, 11.5 %). The majority of the MSSs were composed of monomorphous spindle-shaped cells with moderate cytoplasm and hyperchromatic nuclei with inconspicuous, small nucleoli (Fig. 2a). The only case of BSS was predominantly composed of oval epithelial nests surrounded by malignant spindle cells (Fig. 2b). Among the three PDSSs, two cases were composed entirely of high-grade spindle-shaped cells (Fig. 2c) resembling malignant peripheral nerve sheath tumor (MPNST), and the remaining one tumor was predominantly composed of large epithelioid cells (Fig. 2d) and was reminiscent of carcinoma or mesothelioma. The mitotic rate ranged from two to 36 mitoses per ten high-power field (HPF)s. Other features included a hemangiopericytoma-like vasculature (76.9 %, 20/26), cystic changes (26.9 %, 7/26), myxoid degeneration (42.3 %, 11/26), necrosis (38.5 %, 10/26), and entrapped pneumocytes (3.8 %, 1/26) (Fig. 3a). Grading was performed on 24 samples with sufficient material. The tumors were classified as FNCLCC 2 (15/24, 62.5 %) and FNCLCC 3 (9/24, 37.5 %). The FNCLCC 3 tumors consisted of three PDSSs and six MSSs.

**Table 3:** Histopathologic and immunohistochemical features of 26 PPMSSs

Case NO.	Subtype	Grade (FNCLCC)	Mitotic Rate (/10HPF)	TLE1	ki-67	EMA	CD99	CK7	Bcl2	cytokeratin	CD34	S-100
1	MFSS	2	7	-	50 %	-	-	-	+	+	-	-
2	MFSS	3	36	2+	35 %	+	+	+	+	+	ND	-
3	MFSS	2	2	3+	6 %	+	-	+	+	+	ND	-
4	PDSS	3	12	2+	ND	+	+	-	+	+	-	+
5	MFSS	NA	2/3HPF	2+	ND	+	+	ND	ND	+	-	-
6	MFSS	3	12	3+	4 %	+	-	-	+	+	ND	-
7	MFSS	2	14	2+	35 %	+	+	+	+	+	-	+
8	MFSS	2	2	3+	10 %	+	-	-	+	+	-	-
9	MFSS	3	2	1+	10 %	+	-	+	+	-	+	-
10	MFSS	2	9	-	ND	+	+	-	+	-	-	-
11	MFSS	2	8	2+	25 %	+	-	+	+	-	ND	+
12	MFSS	2	6	ND	30 %	+	+	ND	-	+	**-**	**-**
13	MFSS	2	4	1+	20 %	+	+	+	+	-	-	ND
14	MFSS	2	5	2+	25 %	+	+	+	+	+	-	-
15	MFSS	2	4	2+	60 %	+	-	+	+	+	-	ND
16	MFSS	NA	2/8HPF	3+	40 %	+	+	+	+	-	-	-
17	PDSS	3	21	3+	40 %	+	+	-	+	-	-	-
18	MFSS	2	17	1+	30 %	+	-	ND	ND	+	-	-
19	MFSS	3	12	3+	75 %	+	+	-	+	-	-	-
20	MFSS	2	8	3+	20 %	-	+	-	+	+	-	+
21	MFSS	2	6	2+	35 %	-	ND	ND	+	ND	-	-
22	MFSS	3	21	3+	45 %	+	-	ND	ND	+	-	-
23	MFSS	2	2	2+	45 %	+	-	ND	+	+	-	-
24	MFSS	3	11	3+	55 %	-	-	ND	+	-	-	-
25	BSS	2	4	3+	20 %	+	+	+	+	+	ND	ND
26	PDSS	3	21	ND	20 %	+	+	ND	+	+	ND	+

*Abbreviation*: *NA* not available, *ND* not done, *MFSS* monophasic fibrous synovial sarcoma, *BSS* biphasic synovial sarcoma, *PDSS* poorly differentiated synovial sarcoma, *HPF* high power field, “+” positive, “-” negative

Detailed information regarding microscopic invasion and margins was available in 17 cases. Eight tumors infiltrated into the adjacent tissues, and the remaining nine were free of invasion. The surgical margins were negative in 14 tumors and positive in three tumors. Ten patients underwent lymph node dissections; the eight cases with available information were free of lymph node metastasis (Table 2).

### Immunohistochemical findings

The immunohistochemical results are summarized in Table 3. TLE1 positivity was present in 91.7 % PPMSSs (22/24), with “≥2+” in 79.2 % (19/24) of the tumors (Fig. 3b). In the 39 nonsynovial sarcoma samples, 94.9 % (37/39) were completely negative for TLE1, whereas only two SFTs showed “1+” staining. None of the control tumors showed moderate or strong positivity (“≥2+”) for this marker. The sensitivity and specificity of TLE1 for PPMSS was 91.7 % and 94.9 %, respectively.

Diffuse staining for Bcl-2 and CD99 was present in 95.7 % (22/23) and 56.0 % (14/25) of PPMSSs, respectively. Focal positivity was seen with EMA (84.6 %, 22/26), CK7(55.6 %, 10/18), cytokeratin (AE1/AE3) (68.0 %, 17/25), CD34 (5.0 %,1/20) and S-100 protein (21.7 %, 5/23). A high Ki-67 index (≥10 %) was observed in 91.3 % (21/23) of the tumors.

### FISH and RT-PCR

The molecular results are described in Table 4. By FISH, 25 cases showed *SS18* rearrangement (Fig. 3c). In one of the FISH-positive cases (case 9), at least 60 % of the nuclei contained two paired signals with multiple extra red signals, and loss of green signal (Fig. 3d). One case failed FISH based on absent fluorescent signals; this case (case 2) showed a *SS18-SSX1* fusion on RT-PCR.

**Table 4:** FISH and RT-PCR features of 26 PPMSSs

Case NO.	FISH	RT-PCR	Fusion site of exon	Fusion site of codon
1	+	*SS18-SSX1*	*SS18*:exon 10 *SSX1*:exon 6	*SS18*:codon 410 *SSX1*:codon 111
2	failed	*SS18-SSX1*	*SS18*:exon 10 *SSX1*:exon 6	*SS18*:codon 410 *SSX1*:codon 111
3	+	*SS18-SSX1*	*SS18*:exon 10 *SSX1*:exon 6	*SS18*:codon 410 *SSX1*:codon 111
4	+	*SS18-SSX1*	*SS18*:exon 10 *SSX1*:exon 6	*SS18*:codon 410 *SSX1*:codon 111
5	+	ND	ND	ND
6	+	*SS18-SSX2*	*SS18*:exon 10 *SSX2*:exon 6	*SS18*:codon 410 *SSX2*:codon 111
7	+	*SS18-SSX1*	*SS18*:exon 10 *SSX1*:exon 6	*SS18*:codon 410 *SSX1*:codon 111
8	+	*SS18-SSX1*	*SS18*:exon 10 *SSX1*:exon 6	*SS18*:codon 410 *SSX1*:codon 111
9	+	*SS18-SSX1*	*SS18*:exon 10 *SSX1*:exon 6	*SS18*:codon 410 *SSX1*:codon 111
10	+	ND	ND	ND
11	+	*SS18-SSX1*	*SS18*:exon 10 *SSX1*:exon 6	*SS18*:codon 410 *SSX1*:codon 111
12	+	*SS18-SSX1*	*SS18*:exon 10 *SSX1*:exon 6	*SS18*:codon 410 *SSX1*:codon 111
13	+	*SS18-SSX1*	*SS18*:exon 10 *SSX1*:exon 6	*SS18*:codon 410 *SSX1*:codon 111
14	+	*SS18-SSX1*	*SS18*:exon 10 *SSX1*:exon 6	*SS18*:codon 410 *SSX1*:codon 111
15	+	*SS18-SSX2*	*SS18*:exon 10 *SSX2*:exon 6	*SS18*:codon 410 *SSX2*:codon 111
16	+	*SS18-SSX1*	*SS18*:exon 10 *SSX1*:exon 6	*SS18*:codon 410 *SSX1*:codon 111
17	+	-	-	-
18	+	*SS18-SSX2,* variant	*SS18*:exon 9 *SSX2*:exon 5	*SS18*:codon 366 *SSX2*:codon 94
19	+	*SS18-SSX2*	*SS18*:exon 10 *SSX2*:exon 6	*SS18*:codon 410 *SSX2*:codon 111
20	+	*SS18-SSX2,* variant	*SS18*:exon 9 *SSX2*:exon 5	*SS18*:codon 366 *SSX2*:codon 94
21	+	*SS18-SSX2*	*SS18*:exon 10 *SSX2*:exon 6	*SS18*:codon 410 *SSX2*:codon 111
22	+	*SS18-SSX1*	*SS18*:exon 10 *SSX1*:exon 6	*SS18*:codon 410 *SSX1*:codon 111
23	+	ND	ND	ND
24	+	*SS18-SSX4*	failed	failed
25	+	*SS18-SSX1*	*SS18*:exon 10 *SSX1*:exon 6	*SS18*:codon 410 *SSX1*:codon 111
26	+	*SS18-SSX1*	*SS18*:exon 10 *SSX1*:exon 6	*SS18*:codon 410 *SSX1*:codon 111

*ND* not done, “+” positive, “-” negative

**Fig. 4:**
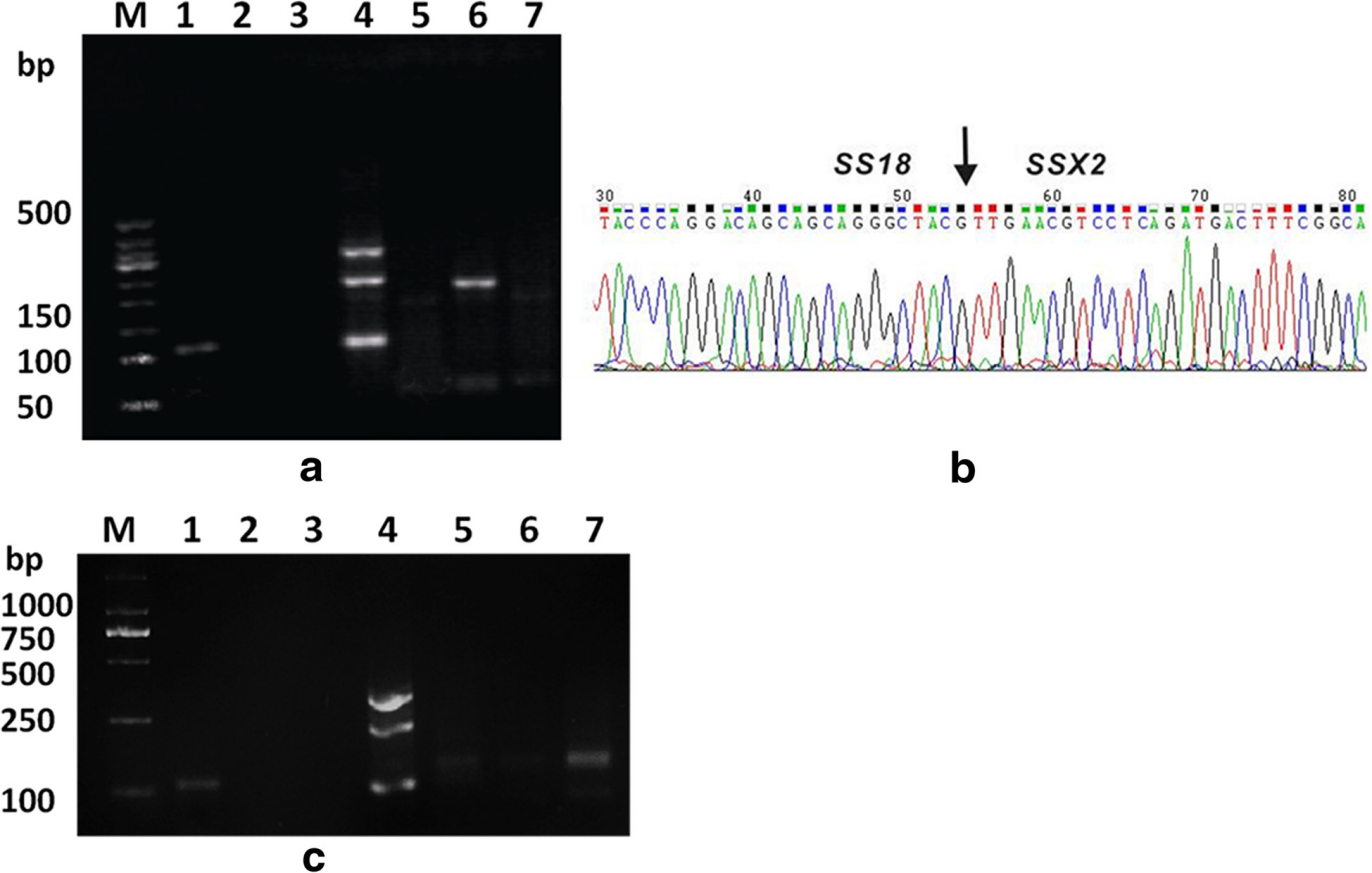
RT-PCR for *SS18-SSX* gene fusions*.* Rare fusion site for *SS18-SSX2* fusion transcript (case 18) (**a** and **b**). **a** Gel electrophoresis of the RT-PCR product. Lane M, size marker; Lane 1, positive control; Lane 2, negative control; Lane 3, blank control; Lane 4, internal control (GAPDH); Lane 5, *SS18-SSX1*; Lane 6, *SS18-SSX2*; Lane 7, *SS18-SSX4.* **b** Partial nucleotide sequence of the *SS18-SSX2* fusion transcript; the fusion sites (*arrow*) are involved in exon 9 of the *SS18* gene (codon 366) and exon 5 of the *SSX2* gene (codon 94). **c** *SS18-SSX4* (case 24). The gel-electrophoresis demonstrates obvious *SS18-SSX4*, with clean lanes of *SS18-SSX1* and *SS18-SSX2*

Of the 23 cases subjected to RT-PCR, 22 were positive for *SS18-SSX* gene fusion, whereas the remaining one was negative (case 17, positive by FISH). The fusion transcripts included *SS18-SSX1* (15/22, 68.2 %), *SS18-SSX2* (6/22, 27.3 %)*,* and *SS18-SSX4* (1/22, 4.5 %) fusions. DNA sequencing showed that the fusion sites of all *SS18-SSX1* and the four classical *SS18-SSX2* tumors were involved in exon 10 of the *SS18* gene (codon 410) and exon 6 of the *SSX1* or *SSX2* genes (codon 111), which is typical of the ordinary fusion site of synovial sarcoma [23]. The remaining two *SS18-SSX2* tumors (cases 18 and 20) showed involvement of exon 9 of the *SS18* gene (codon 366) and exon 5 of the *SSX2* gene (codon 94) (Fig. 4a and b), which has not been described in the literatures. *SS18-SSX4* is shown in the gel-electrophoresis (Fig. 4c), whereas the lanes of *SS18-SSX1* and *SS18-SSX2* were clear. However, sequencing of this rare sample failed.

### Survival analysis

Relapse and survival information are summarized in Table 2. Follow-up data were available for 73.1 % of cases (19/26) with a median follow-up time of 12.0 months; these data included 15 patients with localized disease and four patients with tumor metastasis at presentation. Among the 15 patients with localized disease, ten (66.7 %) suffered disease progression two to 51 months after diagnosis (median, 8.5 months; mean, 15.9 months), including six patients with local recurrence and five patients with metastasis. Thirteen patients (68.4 %) died of the disease within 51 months. Four patients (21.1 %) were alive with the disease. Two (10.5 %) were disease free with a short follow-up time. No patients lived without evidence of disease for more than 24 months. In the entire cohort, the median survival time (MST) was 14.5 months (range 1–52 months). The 2-year disease-specific survival was 27.7 %.

Log-rank analyses on the prognostic parameters were as follows: gender (χ^2^ = 1.25, *p* = 0.246 > 0.05), age (≥37 vs. <37; χ^2^ = 0.064, *p* = 0.800 > 0.05), tumor size (≥5 cm vs. <5 cm; χ^2^ = 0.56, *p* = 0.454 > 0.05), FNCLCC grade (χ^2^ = 0.17, *p* = 0.685 > 0.05), fusion gene types (*SS18-SSX1* vs. *SS18-SSX2*; χ^2^ = 0.10, *p* = 0.756 > 0.05), mitotic rate (≥10/10 HPFs vs. <10/10 HPFs; χ^2^ = 0.01, *p* = 0.925 > 0.05), tumor resection (yes vs. no; χ^2^ = 5.13, *p* = 0.024 < 0.05) and tumor residual status (yes vs. no; χ^2^ = 8.55, *p* = 0.004 < 0.05). Surgical resection and no residual tumor were associated with significantly improved overall survival (OS). No significant difference was observed between two sides of the other survival factors.

## Discussion

PPMSSs are extremely rare; however, according to our series, SS accounted for 14.7 % of all primary sarcoma in pleuropulmonary and mediastinal area and seems to be the most common primary sarcoma type in this region. A preference for location at the lung (76.9 %) was observed, which was similar to the findings of prior studies [11, 14, 15]. Additionally, lung appears to be the most common organ-based location for SS [24]. Therefore, more attention should be paid to this peculiar entity.

In this study, the tumors displayed a male predominance (17 males and nine females; ratio, 1.9:1.0), which is in agreement with a previous series (ratio, 2.0:1.0) [9]. In contrast, a study from Japan showed a female dominance (ratio, 0.6:1.0) [12]. However, there was no obvious sex predominance in the majority of previous studies [10, 11, 13–17]. Our findings demonstrated that PPMSSs often develop in young to middle-aged adults (median, 36.5 years; mean, 37.8 years), older than that of the soft tissue counterpart (median, 34.0 years [25]). Historical series showed similar findings. Chan et al. [26] observed that SS in older people were more likely to occur in unusual regions. The mean age of patients with PPMSS in this study was somewhat lower than that reported in the largest series [14] (37.8 years vs. 42 years) and much lower than that of the Japanese series [12] (37.8 years vs. 58 years). Cough, chest pain and dyspnea were the common presenting signs and symptoms in this study, whereas a few cases were detected incidentally. In our study, only a single patient developed pneumothorax; however, in the series of Cummings et al. [27], pneumothorax appeared to be a frequent event in cystic pulmonary SSs, partly because of the superficial location of the masses.

Radiographically, PPMSSs are typically homogenous and well-defined, and ipsilateral pleural effusions could be observed in most cases. On CT, the tumors in this study could present with sharp (66.7 %) or ill-defined (33.3 %) margins. The tumors were manifested by heterogeneous enhancement and rarely by homogeneous enhancement, which was similar to the findings of earlier studies [16–19] and their soft tissue counterparts [28]. Calcification was absent in our series and in most previous series although it is a common feature of soft tissue SSs [28]. A mediastinal shift was usually observed in the patients with very large masses, and ipsilateral pleural effusions are also a common sign. Generally, the “triple signal” pattern (hypointense, isointense, and hyperintense) was a frequent event on T2-weighted magnetic resonance imaging [17].

Grossly, the majority of tumors (72.2 %) in this study were well circumscribed, and the tumors exhibited a variety of hemorrhage, necrosis, and cystic changes, which were similar to those seen in previous reported SSs [14, 17].

Histologically, MSS (84.6 %) was the main subtype, followed by PDSS (11.5 %) and BSS (3.8 %); these features are similar to the prior PPMSS series [11–16] and soft series [25, 28]. In this series, microscopic calcification was absent, and some previous series also showed that calcification appears to be much less common than in soft tissue lesions (15 % vs. 30 %) [9, 11, 13, 14, 28].

The proportions of FNCLCC 2 (62.5 %)) and FNCLCC 3 (37.5 %) in our series were similar to the largest series (75.8 % and 24.2 %, respectively) [14] and most soft tissue SSs (69.0 ~ 74.0 % and 26.0 ~ 31.0 %, respectively) [29, 30]. However, the proportion of FNCLCC 3 in Begueret’s study [13] was as high as 70 %. The explanation for the difference between the results of Begueret et al. [13] and other authors requires further studies.

TLE1 has recently emerged as a new marker for the diagnosis of SS [15, 21]. However, the diagnostic value of this antibody remains controversial [31]. In this series, TLE1 demonstrated high sensitivity (91.7 %) and specificity (94.9 %) for PPMSSs. Although some SFTs showed weak positivity for TLE1, none of the nonsynovial sarcomas were moderately or strongly reactive for this marker. Therefore, TLE1 could be used as a screening marker prior to the detection of t(X;18)(p11.2;q11.2) [32].

A high Ki-67 index (≥10 %) in our study occurred in 91.3 %, which was higher than in the prior Japanese series [12] (45.5 %) and in most soft tissue counterparts [22] (26.5 ~ 86.0 %). Ki-67 has been suggested as a prognostic factor in synovial sarcoma [22], and the poor prognosis might be partly explained by this high Ki-67 staining in our series.

In this series, the fusion transcripts included *SS18-SSX1* (68.2 %), *SS18-SSX2* (27.3 %)*,* and *SS18-SSX4* (4.5 %) fusions, and the proportion of *SS18-SSX1* and *SS18-SSX2* fusions were similar to that in previous studies [12–14]. However, some different findings were as follows. First, we found a single case harboring a *SS18-SSX4* fusion transcript. This fusion presents in only 1–2 % of SSs [25], and to the best of our knowledge, this extremely rare fusion has not been previously reported in PPMSS before. This case was classified as MSS (FNCLCC 3). Local recurrence occurred 9 months after the tumor excision and postoperative radiotherapy. Second, two cases harboring rare fusion site of *SS18-SSX2* fusion transcript were identified and this fusion site has not been previously reported in SSs. Further studies are needed to determine whether the variant fusion site is specific to PPMSS. Third, one case was found to harbor a complex pattern by FISH analysis. This case exhibited two paired signals with multiple extra red signals and loss of green signal, which are suggestive of a cryptic rearrangement of the 5’ region of the *SS18* gene; this case was proven to be *SS18-SSX1* by subsequent RT-PCR. This finding has not been described in PPMSSs, although it has been reported in soft tissue SSs [33, 34], and the presence of aneuploidy or amplificon of chromosome 18q might account for this event. This case was MSS (FNCLCC 3) and was the only case with massive necrosis in our series. This patient developed a local recurrence only 6 months after the tumor excision and died 12 months after diagnosis. However, more samples are needed to further analyze the correlation of prognosis and the special genetic changes.

The differential diagnosis of PPMSS is broad and might be very challenging, especially in small biopsy samples. This neoplasm must be differentiated from secondary SS and other primary spindle cell neoplasms in these locations.

The intrathoracic area is an extraordinarily rare location for SS. For example, in our Medical Centre, among the 56 cases of SS in this location, 50 % were metastatic tumors. Therefore, the possibility of a secondary SS should always be excluded. In this series, detail clinical information ruled out the possibility of metastatic lesions arising from other sites.

Sarcomatoid carcinoma could have a conspicuous spindle cell component and show focal positivity for only epithelial markers, simulating SS. SS could also display epithelioid features and might be confused with carcinomas or mesothelioma. Therefore, sarcomatoid carcinoma and mesothelioma should always be the primary consideration in the differential diagnosis of SS. However, carcinoma and mesothelioma could be distinguished from SS in several aspects. First, the radiologic findings usually show an infiltrative border for carcinoma and a diffuse growth pattern for mesothelioma, whereas PPMSS typically appears as a well-circumscribed mass with uniform opacity [17]. Second, sarcomatoid carcinoma and mesothelioma are usually more pleomorphic than SS, and extensive sampling of the lesion could help identify an underlying histologic subtype of lung carcinoma. Third, neither sarcomatoid carcinoma nor mesothelioma exhibit strong reactivity for TLE1 in this study and Lino-Silva et al. [15] although Matsuyama et al. [35] found TLE1 staining in 69 % of mesotheliomas. Fourth, identification of t(X;18)(p11.2;q11.2) could be invaluable for extremely challenging cases.

Type A thymomas and type I pleuropulmonary blastomas could share clinical and morphological features with PPMSS. However, type I pleuropulmonary blastomas typically develop in infants and during early childhood. In contrast, PPMSSs typically occur in older patients [13, 14]. Careful morphologic inspection and ancillary immunohistochemical stains and molecular studies are helpful in distinguishing between these lesions.

Intrathoracic SFTs might share some morphologic features with PPMSSs. SSs always do not exhibit diffuse reactivity for CD34 [2], whereas SFT usually do not show strong positivity for TLE1, as seen in the this study. SFTs are negative for t(X;18)(p11.2;q11.2) and have been shown to harbor a characteristic *NAB2-STAT6* fusion gene [36]. Very recently, STAT6 has proven to be a highly sensitive and specific marker for the diagnosis of SFT [37].

MPNST could closely resembles SS and appears to be the most challenging tumor type in the differential diagnosis of SS in any location. A full panel of markers including TLE1 and molecular genetic testing for t(X;18)(p11.2;q11.2) could be useful for distinguishing between the two tumors.

Poorly differentiated SS might be almost entirely poorly differentiated with uniform small round cell morphology, resembling Ewing sarcoma. Extensive sampling and careful inspection are required to identify any accompanying more typical areas of synovial sarcoma, which play an important role in establishing the diagnosis. Immunostaining for TLE1 and identification of *SS18-SSX* fusion instead of *EWSR1-FLI-1/EWSR1-ERG* [38] could help to confirm the diagnosis.

The preferred treatment of SSs is radical resection combined with radiotherapy and or chemotherapy, which could remarkably or partly decrease the recurrent or metastatic rate [39, 40]. In our series, 20 patients (76.9 %) underwent lobectomy or tumor resection, and 82.4 % of these patients obtained negative margins. Seven of these surgical patients (35 %) underwent adjuvant radiotherapy or/and chemotherapy. Several previous PPMSSs series have described the treatment for PPMSS [9–13, 16]; however, few previous studies analyzed the correlation between treatment and survival. Our results indicated that effective excision of the tumors with negative margins could significantly improve survival (*p* = 0.004), and tumor resection is important when available (*p* = 0.024).

According to the current study, PPMSSs appear to be more aggressive than the soft tissue counterparts (MST, 14.5 months vs. 52 months [41]); these results are similar to the results of previous studies. The prognosis of PPMSSs in this study appear more unfavorable than in prior series of PPMSSs (MST, 14.5 months vs. 30 ~ 50 months) [11–13, 15, 16]. None of the subjects in our series survived for more than 5 years, and none of the subjects lived with no evidence of disease for more than 2 years; the 5-year survival rate in prior series was approximately 30 % [13], and it could reach 50 % in soft tissue SSs [11, 42]. The dismal prognosis of PPMSSs might partly be due to later presentation and difficulties in obtaining negative surgical margins.

In terms of prognostic factors, age (better in young patients), tumor size (<5 cm), mitotic rate (<10/10HPF), FNCLCC (low grade), status of the surgical margins (negative), and *SS18-SSX* type (*SS18-SSX2*) were reported to be associated with better survival in soft tissue SSs [25, 30]. We found tumor resection (*p* = 0.024) and no residual tumor (*p* = 0.004) were significantly associated with better survival. The other factors had no effect on survival.

## Conclusions

Here, we present the clinicopathological and molecular features of 26 t(X;18)(p11.2;q11.2)-positive PPMSSs. These tumors seem to be more aggressive than the soft tissue counterparts and more aggressive than prior PPMSSs series. Extensive surgical resection of the tumor and more effective adjuvant therapy should be advocated. The diagnosis of PPMSS might be challenging. A combination of clinical studies, careful morphologic analysis, and a full panel of immunomarkers including TLE1 and genetic studies is helpful in confirming the diagnosis.

## Abbreviations

BSS, biphasic synovial sarcoma; CT, computed tomography; FFPE, formalin-fixed paraffin-embedded; FISH, fluorescence in situ hybridization; FNCLCC, French Federation of Cancer Centers; HPF, high-power field; MPNST, malignant peripheral nerve sheath tumor; MSS, monophasic synovial sarcoma; MST, median survival time; OS, overall survival.; PDSS, poorly differentiated synovial sarcoma; PPMSS, primary pleuropulmonary and mediastinal synovial sarcoma; RT-PCR, reverse transcription polymerase chain reaction; SFT, solitary fibrous tumor; SS, synovial sarcoma
